# Aligning Standards Communities for Omics Biodiversity Data: Sustainable Darwin Core-MIxS Interoperability

**DOI:** 10.3897/BDJ.11.e112420

**Published:** 2023-10-03

**Authors:** Raïssa Meyer, Ward Appeltans, William D. Duncan, Mariya Dimitrova, Yi-Ming Gan, Thomas Stjernegaard Jeppesen, Christopher Mungall, Deborah L Paul, Pieter Provoost, Tim Robertson, Lynn Schriml, Saara Suominen, Ramona Walls, Maxime Sweetlove, Visotheary Ung, Anton Van de Putte, Elycia Wallis, John Wieczorek, Pier Luigi Buttigieg

**Affiliations:** 1 Alfred Wegener Institute, Helmholtz Centre for Polar and Marine Reserach, Bremerhaven, Germany Alfred Wegener Institute, Helmholtz Centre for Polar and Marine Reserach Bremerhaven Germany; 2 Max Planck Institute for Marine Microbiology, Bremen, Germany Max Planck Institute for Marine Microbiology Bremen Germany; 3 University of Bremen, Faculty of Geosciences, Bremen, Germany University of Bremen, Faculty of Geosciences Bremen Germany; 4 Intergovernmental Oceanographic Commission of UNESCO, Ocean Biodiversity Information System (OBIS), Oostende, Belgium Intergovernmental Oceanographic Commission of UNESCO, Ocean Biodiversity Information System (OBIS) Oostende Belgium; 5 University of Florida, Gainesville, United States of America University of Florida Gainesville United States of America; 6 Bulgarian Academy of Sciences, Sofia, Bulgaria Bulgarian Academy of Sciences Sofia Bulgaria; 7 Pensoft Publishers, Sofia, Bulgaria Pensoft Publishers Sofia Bulgaria; 8 Royal Belgian Institute of Natural Sciences, Brussels, Belgium Royal Belgian Institute of Natural Sciences Brussels Belgium; 9 Global Biodiversity Information Facility (GBIF), Copenhagen, Denmark Global Biodiversity Information Facility (GBIF) Copenhagen Denmark; 10 Lawrence Berkeley National Laboratory, National Microbiome Data Collaborative (NMDC), Berkeley, United States of America Lawrence Berkeley National Laboratory, National Microbiome Data Collaborative (NMDC) Berkeley United States of America; 11 University of Illinois, Illinois Natural History Survey, Species File Group, Champaign-Urbana, United States of America University of Illinois, Illinois Natural History Survey, Species File Group Champaign-Urbana United States of America; 12 Department of Epidemiology and Public Health, University of Maryland School of Medicine, Baltimore, MD, USA, Baltimore, United States of America Department of Epidemiology and Public Health, University of Maryland School of Medicine, Baltimore, MD, USA Baltimore United States of America; 13 Critical Path Institute, Tucson, United States of America Critical Path Institute Tucson United States of America; 14 UMR 7205 CNRS-MNHN-SU-EPHE-UA, Paris, France UMR 7205 CNRS-MNHN-SU-EPHE-UA Paris France; 15 Royal Belgian Institute of Natural Sciences, Université Libre de Bruxelles, Brussels, Belgium Royal Belgian Institute of Natural Sciences, Université Libre de Bruxelles Brussels Belgium; 16 Atlas of Living Australia, CSIRO, Melbourne, Australia Atlas of Living Australia, CSIRO Melbourne Australia; 17 University of California, Berkeley, United States of America University of California Berkeley United States of America; 18 Helmholtz Metadata Collaboration, GEOMAR Helmholtz Centre for Ocean Research, Kiel, Germany Helmholtz Metadata Collaboration, GEOMAR Helmholtz Centre for Ocean Research Kiel Germany

**Keywords:** microbiome, eDNA, biodiversity, information standards, omics, metadata, harmonization, FAIR, MIxS, Darwin Core

## Abstract

The standardization of data, encompassing both primary and contextual information (metadata), plays a pivotal role in facilitating data (re-)use, integration, and knowledge generation. However, the biodiversity and omics communities, converging on omics biodiversity data, have historically developed and adopted their own distinct standards, hindering effective (meta)data integration and collaboration.

In response to this challenge, the Task Group (TG) for Sustainable DwC-MIxS Interoperability was established. Convening experts from the Biodiversity Information Standards (TDWG) and the Genomic Standards Consortium (GSC) alongside external stakeholders, the TG aimed to promote sustainable interoperability between the Minimum Information about any (x) Sequence (MIxS) and Darwin Core (DwC) specifications.

To achieve this goal, the TG utilized the Simple Standard for Sharing Ontology Mappings (SSSOM) to create a comprehensive mapping of DwC keys to MIxS keys. This mapping, combined with the development of the MIxS-DwC extension, enables the incorporation of MIxS core terms into DwC-compliant metadata records, facilitating seamless data exchange between MIxS and DwC user communities.

Through the implementation of this translation layer, data produced in either MIxS- or DwC-compliant formats can now be efficiently brokered, breaking down silos and fostering closer collaboration between the biodiversity and omics communities. To ensure its sustainability and lasting impact, TDWG and GSC have both signed a Memorandum of Understanding (MoU) on creating a continuous model to synchronize their standards. These achievements mark a significant step forward in enhancing data sharing and utilization across domains, thereby unlocking new opportunities for scientific discovery and advancement.

## Introduction

In recent years, the field of biodiversity research has witnessed rapid growth in data acquisition, further driven by the increasing application of omics technologies (e.g. metagenomics or metatranscriptomics) in biodiversity assessments. However, the sheer volume and heterogeneity of biodiversity data pose significant challenges to effective data integration and reuse, and to FAIR Wilkinson et al. 2016 management. To address these challenges, both the biodiversity and the omics research communities have recognized the urgent need for (meta)data standards.

The Biodiversity Information Standards (TDWG; https://www.tdwg.org/) group and the Genomic Standards Consortium (GSC; https://www.gensc.org/) Field et al. 2011 have emerged as de facto (meta)data standards authorities in the biodiversity and omics domains, respectively. The former’s scope spans biodiversity data at large, while the latter focuses on genomic, and then multi-omic data and metadata such as lab protocols or chemical/physical measurements. Their activities, technologies, and management structures have been largely parallel, with some notable exceptions catalyzed through joint interest groups such as the Genomic Biodiversity Working Group (GBWG; https://www.tdwg.org/community/gbwg/).

The overlap of TDWG and the GSC in multi-omic biodiversity data is an opportunity to begin sustainable convergence of the (meta)data standards these organizations maintain. Most notable among these are the Darwin Core (DwC; https://dwc.tdwg.org/) Wieczorek et al. 2012 and the Minimal Information about any (x) Sequence (MIxS; https://www.gensc.org/pages/standards-intro.html) Yilmaz et al. 2011 specifications.

These two (meta)data standards have co-existed for a number of years, but adoption of one or the other is still leading to the siloing of information and a resulting lack of sustained interoperability between systems such as those of the International Nucleotide Sequence Database Collaboration (INSDC; https://insdc.org), and of the Ocean Biodiversity Information System (OBIS; https://obis.org) or the Global Biodiversity Information Facility (GBIF; https://www.gbif.org/). Meanwhile, some of these stakeholders are creating bespoke/local interpretations of DwC/MIxS mappings, which may further silo the digital holdings of the omic biodiversity community.

In the Sustainable DwC-MIxS Interoperability Task Group (TG), we brought together experts to build semantically precise and sustained interoperability between TDWG’s DwC standard, and the MIxS checklist from the GSC.

We aim to consolidate previous work on this issue Tuama et al. 2012 into a stable, operational, and more authoritative cross-embedding of both de facto standards. This is becoming an urgent need by international efforts moving into the domain of omically-enabled biodiversity research and operations.

A key motivation for consolidation is to ensure the "digital health" efforts leveraging the immense interest in using omic technologies to observe life in the oceans under the UN Decade of Ocean Science for Sustainable Development (2021-2030; https://oceandecade.org/). Stakeholders rallying around this global call either use both standards or wish to collaborate across them as part of the Decade's digital strategy (see Section 2.5. Data, information, and digital knowledge management in the Implementation Plan UNESCO-IOC 2021). The organizations that are the custodians of these standards need to agree on a functional and stable interoperation solution. Otherwise, there will be increasing confusion and digital overhead in using omics biodiversity data to deepen our understanding of the marine ecosystem, increase our knowledge about drivers of change, and consequences of change, and inform policy decision-makers.

This TG aimed to produce an approach to sustainably align the MIxS and DwC (meta)data specifications to enhance more efficient and interoperable exchange across their user communities. In the following, we present our report on building sustainable interoperability between DwC and MIxS, including a mapping between DwC and MIxS, a MIxS extension to DwC, as well as a Memorandum of Understanding (MoU) between TDWG and the GSC.

## A note on terminology

MIxS and DwC both use terms (strings associated with a meaning) to identify elements of data structures. That is, terms (such as “elevation”) are used to identify the intended meaning of, for example, 1) the attributes/columns in tabular data or 2) keys in key-value pairs. Both specifications provide metadata about their terms, clarifying their intended meaning and the expected values that should be associated with them once they are cast in a data structure (i.e., values in table cells, or values in key-value pairs).

Typically, in both MIxS and DwC data exchanges between human agents, (meta)data is arranged in spreadsheets or tabular form. The terms are thus used as attribute names/column headers. When archived in the INDSC (MIxS) and/or GBIF/OBIS (DwC), terms are rendered as keys in key-value pairs. Below, for precision, we default to the usage of “key” (e.g., “temperature”) and its associated “value” (e.g., “18”*^1^).*^2^

## Glossary

Table 1

## Approach

### Mapping

Simple Standard for Sharing Ontology Mappings (SSSOM; https://mapping-commons.github.io/sssom/home/) Matentzoglu et al. 2022 provides a list of minimal and standard metadata elements. These are used in combination with standard predicate terms, such as the Simple Knowledge Organization System (SKOS; https://www.w3.org/TR/skos-reference/) terms to provide mappings between terms in differing terminologies (or ontologies).

We performed a comprehensive mapping from DwC to MIxS, capturing differences in both semantics and syntax between corresponding keys using the format of the SSSOM.

The semantic mapping was based on the minimal and standard set of metadata elements provided by SSSOM, in combination with the relevant SKOS predicates.

As the SSSOM standard set of metadata elements does not yet*^6^ include means to capture information about the syntactic alignment of terms*^7^, we expanded the list of metadata elements to additionally capture information on the syntactic alignment of mapped terms (see Table 2). The additional metadata elements were added to our syntactic mapping document in replacement of the semantic mapping metadata attributes.

Table 2

To facilitate the mapping process during our working period, we additionally added further metadata elements to capture definitions and value syntax (see Table 3). This working document is also available through our GitHub repository*^8^. This is a secondary output which might be of relevance for future TGs performing mappings between metadata standards.

Table 3

For each mapping, group consensus was reached through a combination of structured discussions in the GitHub issue tracker and online video-chat meetings. Mappings can be found in the TDWG/GBWG GitHub repository, with related discussions captured on the issue tracker.

The SSSOM compliance of the mapping products was validated by Chris Mungall*^9^ and Harshad Hegde*^10^.

The aims of the mapping process were to provide:

- qualifications, which explain if discrepancies in semantics or syntax are to be expected and suggest how these can be resolved.
- DwC and MIxS keys identified by IRIs as opposed to labels.
- semantic mappings between DwC and MIxS keys following the SSSOM specification, using SKOS predicates (e.g., SKOS:exactMatch).
- semantic predicates and comments on the semantic mapping in the SSSOM matrix.
- augmentation of the SSSOM matrix to also include information on the level of syntactic compatibility. For example, the DWC key *decimalLatitude* expects values in the key-value pair to be decimals, whereas the MIxS key *lat_lon* does not.

### Extension

Darwin Core Archives are generally built on a combination of a core CSV file and zero or more extension CSV files. The schemas of the core and extensions are defined by XML documents maintained in the GBIF GitHub repository for machine-readable resources (https://github.com/gbif/rs.gbif.org). Core files act as the primary focus of a data set (e.g., Occurrences of organisms in nature), while the extensions add information relevant for specific uses (e.g., the proposed MIxS extension). The MIxS extension contains the list of keys that are orthogonal (have no equivalent mappings) to keys in the Darwin Core standard. Being orthogonal and defined by GSC, the keys in the extension are identified by IRIs from a namespace (fully qualified namespace [https://w3id.org/mixs/] became available with the release of MIxS V6) distinct from that of Darwin Core (http://rs.tdwg.org/dwc/terms/).

This was achieved by 1) documenting the relevant MIxS terms in the XML format specified by GBIF*^11^ and 2) creating vocabulary definitions in the XML format specified by GBIF*^12^ that contain the thesauri for the terms that should have controlled vocabularies.

### Testing

To test technical interoperability and simulate the ingestion of MIxS-compliant metadata into a Darwin Core-based database environment (e.g., OBIS or GBIF), a marine omics dataset Franco et al. 2017 was selected (available in ENA and in the GBIF test environment: Dataset in GBIF, a Sampling Event [scroll down to see taxonomic breakdown], an occurrence [scroll down to see the MIxS data]. This dataset was previously published to GBIF as metadata-only, and represents a typical use case where the community composition of microbes was profiled by high throughput amplicon sequencing of the 16S rRNA gene. This generates microbial occurrences of both known and unknown species that are exclusively based on environmental DNA sequences. These sequences are available under the Bioproject PRJNA335729 on the databases of the International Nucleotide Sequence Database Consortium. The sequence metadata was provided compliant with MIxS v5, and sequences along with corresponding taxonomic annotation were downloaded from MGnify*^13^ in BIOM and FASTA formats and converted to DwC occurrences using a script available on GitHub: https://github.com/thomasstjerne/antarctic-marine-sediments-dwc.

Similar tests were performed using data representing Pico- to Mesoplankton along the 2000 km Salinity Gradient of the Baltic Sea Hu et al. 2016 (datasets in GBIF: 18S: https://doi.org/10.15468/5k9w88, 16S: https://doi.org/10.15468/thsjhr) and data from a study demonstrating how nets mounted on rooftops of cars (car nets) and DNA metabarcoding can be applied to sample flying insect richness and diversity across large spatial scales within a limited time period Svenningsen et al. 2021 (https://doi.org/10.5061/dryad.6q573n5z5, test dataset in GBIF https://www.gbif-uat.org/dataset/d6cb82c9-1194-4a80-bf4d-f7f4b041b5d2) using the GBIF Integrated Publishing Toolkit (IPT).

We were able to successfully ingest the data into GBIF's user agreement test environment (www.gbif-uat.org). These test cases show it is possible for omics data to be incorporated along human observation-based occurrence datasets using data processing by MGnify. This advancement is especially relevant for microbial groups, some of which are only known from environmental DNA (eDNA) sequences. It opens up new opportunities to include the vast biodiversity of micro eukaryotes, Bacteria, and Archaea in repositories that up to now have been dominated by plants and animals.

Additionally, OBIS will be working on a first test case of the DNA-derived data extension utilizing Autonomous Reef Monitoring Structures (ARMS) datasets (https://doi.org/10.3389/fmars.2020.572680), which will link occurrences derived from genetic samples, morphological identifications and photographic evidence to each sampling device. To facilitate the addition of sequencing datasets to the database, OBIS is also developing a bioinformatics pipeline, which will output a dataset formatted to the DwC-A including the MIxS extension.

As of Sep. 1st 2023, 16,612,814 Occurrences distributed across 52 datasets have been published to the GBIF production environment and OBIS holds 23 million records/sequences from 36 datasets utilising the GBIF/OBIS variant of the MIxS DwC extension*^14^.

### Community feedback

This TG has solicited and incorporated feedback from the GSC steering group and TDWG executive committee prior to the signing of the Memorandum of Understanding. We welcome feedback from users and implementers of the mapping and extension upon the publishing of this paper. Please share your feedback through the GBWG GitHub issue tracker, using the label "DwC-MIxS feedback V2.1.0".

### Memorandum of Understanding

To ensure that our mapping and approach are integrated into the procedures and workflows of both TDWG and the GSC, we drafted and circulated a Memorandum of Understanding (MoU; see Outcomes) to the executive bodies of each organization. This MoU has been signed in October 2022. It incorporates processes sustaining and furthering interoperability between these specifications and organizations. It is in this way, we hope that the work of our TG can lay the foundation for ever closer alignment, ultimately allowing precise machine-to-machine translation of metadata using GSC and TDWG specifications.

### Ensuring sustainability

GitHub releases of new versions of either DwC or GSC shall trigger a notification to the maintainers of the mapping created by this TG, who will review the new release and update the mapping if needed. As both standards have a release approximately annually, we estimate that long-term maintenance should require approximately 10-30 combined person-hours for mapping review per year, plus review by the TDWG DwC Maintenance Group and the GSC Compliance and Interoperability Group (CIG), each of which can be accomplished as part of one of their regular monthly meetings.

As part of the MOU, both GSC and TDWG have agreed to provide personnel to maintain this mapping in perpetuity and to provide ongoing development to automate the mapping process as possible.

- **DwC release process**: TDWG has an official process for the maintenance of standards embodied in the Vocabulary Maintenance Standard (http://www.tdwg.org/standards/642) and documented in the Vocabulary Maintenance Specification (https://github.com/tdwg/vocab/blob/master/vms/maintenance-specification.md). The Darwin Core Maintenance Group (https://www.tdwg.org/community/dwc/) is responsible for the maintenance and evolution of the standard, including extensions to it, of which MIxS would be one. Updates to the standard result in releases on GitHub (https://github.com/tdwg/dwc/releases), which are backed up on Zenodo. GBIF maintains a repository (https://github.com/gbif/rs.gbif.org/tree/master/extension) of the production versions of the Darwin Core Archive extension XML files which are available to be used to create Darwin Core Archives using the GBIF Integrated Publishing Toolkit (IPT, https://www.gbif.org/ipt).
- **GSC release process**: Premiering with the release of MIxS V6, the GSC has a workflow in place on GitHub which automatically builds new versions of the standards from code, releases stable versions, and backs them up on Zenodo. The normal release cycle for MIxS is about 1 time per year, but with the new release technology, there may be minor releases during the year. The minor releases will always be backward compatible with their major releases and will only include the addition of new terms. Furthermore, new keys can be created for MIxS between releases and be approved as individual keys with a stable URI, but not be considered part of an official MIxS release. This allows the rapid minting of keys while still providing time for a thorough review before changing official releases.

The next update of the mapping is expected before the end of the year with the new release of DwC around the MaterialEntity developments*^15^ and the release of MIxS v6.2, which is currently under development.

## Outcomes

### Mapping

**Note**: The TG developed this mapping based on MIxS v5, the identifiers, however, are based on those noted in the working document preceding the MIxS v6 release*^17^, which were later released with MIxS v6.

Following our mapping approach (Approach: Mapping), we mapped 32 DwC keys to 12 MIxS keys. Our resulting SSSOM records are accessible through the GBWG DwC-MIxS GitHub repository.*^18^ As detailed below (see Recommendations for using the SSSOM mapping matrix and Approach: Mapping), we created three SSSOM records to disaggregate our results:

1. DwC-MIxS_mappingSemantic.tsv*^19^: this record contains mappings based on the meanings of the terms associated with the DwC and MIxS keys.
2. DwC-MIxS_mappingSyntactic.tsv*^20^: this record contains mappings based on the syntactic similarity of the DwC and MIxS keys.
3. DwC-MIxS_mappingSupport.tsv*^21^: this record includes both the semantic in syntactic mappings, as well as the supporting information used to determine both.

### Memorandum of Understanding

In the following section, we include the Memorandum of Understanding (MoU) between TDWG and the GSC.

#### Preamble

The Biodiversity Information Standards (TDWG) group and the Genomic Standards Consortium (GSC) have emerged as de facto (meta)data standards authorities in the biodiversity domain. The former’s scope spans biodiversity data at large, while the latter focuses on genomic, and then multi-omic, data and metadata such as lab protocols or chemical/physical measurements. Their activities, technologies, and management structures have been largely parallel, with some notable exceptions catalyzed through joint interest groups such as the Genomic Biodiversity Working Group (GBWG).

The overlap of TDWG and the GSC in multi-omic biodiversity data is an opportunity to begin sustainable convergence of the (meta)data standards these organizations maintain. Most notably among these, are the Darwin Core (DwC) and the Minimal Information about any (x) Sequence (MIxS) specifications. This memorandum builds on the output of a GBWG task group to propose a solution for sustained mapping and scalable interoperation of both DwC and MIxS. Its goal is to ensure that TDWG and the GSC create a lasting and continuous model to synchronize their standards, eventually promoting full bi-lateral integration.

#### Memorandum

**Recognizing** that both the Biodiversity Information Standards (TDWG) group and the Genomic Standards Consortium (GSC) have established well-adopted and community-driven (meta)data specifications for sequence-based biodiversity data;

**Further recognizing** that users of one standard specification should not have to invest additional effort in independently translating their (meta)data into another;

**It is resolved that**:

- The GSC and TDWG will maintain and endorse an authoritative and machine-readable mapping*^16^ of the fields in their MIxS and DwC (meta)data standard specifications;
- These authoritative mappings (in SSSOM-compliant tab-separated value files) and other digital references will be maintained in the GBWG GitHub repository within the TDWG organization and with TDWG-issued IRIs for the mapping files;
- Further, both organizations will provide bilaterally endorsed reference implementations of how to use their counterpart’s specification in their data structures (e.g., a DwC Archive incorporating fields mapped to MIxS in a DwC extension);
- Any necessary modification of identifiers (URNs, URLs, URIs, IRIs, etc) or other component of a standard issued by one organization which impacts the other should be declared and the particulars agreed upon in documented appendices to this MoU;
- When one specification is updated, the TDWG DwC Maintenance Group and the GSC Compliance and Interoperability Group (CIG) will hold joint sessions to update and validate any mappings and reference implementations to ensure clarity in the multi-omic biodiversity data community.
- The communication channels to communicate updates of either specification will be the MIxS issue tracker*^22^ and the DwC issue tracker*^23^. If any of the terms in the mapping are subject to review, the parties will notify each other through those communication channels.

**Additionally recognizing that** unilateral innovation and research actions will propose and implement alternative mappings and extensions to sequence-based metadata specifications.

**It is further resolved that**:

- Only those modifications which have been reviewed and endorsed by mechanisms bi-laterally convened by TDWG and the GSC will be considered standardized;
- Innovation is still welcome, and both organizations will welcome input and inspiration from application-driven modifications of the base standard.

Signatories:

12. October 2022 Representative of TDWG Executive (Deborah L Paul)

13. October 2022 Representative of the GSC Board (Lynn Schriml)

### DwC extension

#### MIxS-DwC extension

We created a DwC extension*^24^ including the MIxS core keys that do not have a counterpart in DwC, and thus were not included in the mapping (see above). Used in combination with the SSSOM record generated by our TG, the MIxS-DwC extension allows a complete encapsulation of MIxS core in a DwC Archive (modulo some semantic and syntactic mismatches, see Recommendations for Semantic and Syntactic Mapping)

Of the 96 keys contained in MIxS core, we included the 82 terms that were not mapped in the extension.

The TG’s GitHub repository hosts both the list of keys*^25^ and a list of excluded (mapped) keys*^26^. For the keys included in the extension, we have developed a Darwin Core Archive (DwC-A) extension definition in XML*^27^, which provides the standard set of terms that are available, onto which one can map one’s own CSV*^28^.

Following the terms of our MoU draft, this extension will be bilaterally endorsed by the GSC and TDWG to assure users that they are implementing an officially recognized recommendation. The manner in which this is declared (e.g., as a header in the DwC-A reference implementation) will be decided upon by the relevant bodies in the GSC and TDWG.

#### Variations of the MIxS-DwC extension

While the bilaterally endorsed GSC-TDWG extension provides stability, we recognize that the needs of the biodiversity community are more diverse and require more nimble forms of data exchange. In the creation of these more ad hoc extensions, the risk of creating siloed / bespoke data products (and thus reducing global interoperability) is often countered by the practicality of advancing with fewer overheads and at a more rapid pace than standards bodies can be expected to match. Here, without taking a position on the “better” route, we recognize the reality of this scenario.

To demonstrate how metadata fields relevant to sequence-based biodiversity data can relate to the core outputs of this TG, we include a variation of the MIxS-DwC extension - the DNA-derived data extension - developed by GBIF*^29^ as an example of the use (and customization) of the MIxS-DwC extension introduced above. Note again, that this DNA-derived data extension is not built on standards-body synchronization.

The DNA-derived data extension includes all keys of the MIxS-DwC extension, but brings in additional keys necessary to satisfy the exchange needs of the GBIF/OBIS/Atlas of Living Australia (ALA) networks. The additional keys originate primarily from the Minimum Information for Publication of Quantitative Real-Time PCR Experiments (MIQE) recommendation and Global Genome Biodiversity Network (GGBN).

Additionally, the DNA-derived data extension also takes measures to optimize the formatting and machine-readability of keys from MIxS. This stems from the fact that some MIxS key-value pairs are not atomic, i.e., they include multiple values in the same field (e.g., the MIxS key “*pcr_primers*” requires the user to enter a value that is comprised of a string that represents both the forward and reverse primer sequence, separated by a semicolon). This value-level formatting creates a bespoke data structure which then requires custom software or code to parse, limiting interoperability with external systems. Thus, in the case of *pcr_primers*, the DNA-derived data extension uses alternative keys, based on the MIxS key, which are associated with atomic values: *pcr_primer_forward* and *pcr_primer_reverse*. This allows for more efficient and unambiguous data ingestion into search indices, relational databases, or similar solutions, with minimal processing.

We acknowledge that it is a balance for application profiles to both comply with community standard specifications, while also satisfying the needs of the systems using them. To include and represent the evolving needs of the community and applications in existing community standards, we encourage that requests for changes or new keys are directed directly to the GSC*^30^ or TDWG*^31^.

### Recommendations

In the sub-sections below, we offer several recommendations based on the proceedings and outcomes of this TG. We see our TG’s diverse membership and perspectives as a strong model to follow in future work developing or interlinking community standard specifications used by many stakeholders. Through this, operational realities, technical soundness, and policy-level perspectives can be better integrated and built upon.

#### Recommendations for using the SSSOM mapping matrix

The Simple Standard for Sharing Ontology Mappings (SSSOM) offers a framework to represent ontology mappings in a precise way, with a structured way to include rich provenance. For the work of this TG, we have implemented an SSSOM mapping between the DwC standard and the MIxS checklist.

SSSOM provides a minimal set of standard elements for the dissemination of mappings between terms. This helps to ensure a reliable interpretation of mappings and enables sharing and data integration between human and machine agents.

As described in the Recommendations for semantic and syntactic alignment, even closely related MIxS and DwC terms, may have semantic variance, and expect values with different syntax. To manage that variance, we propose extending the list of SSSOM metadata elements to include elements to capture the syntactic mapping (*syntax_predicate_id*, *syntax_comment*; see Approach: Mapping) in addition to the existing semantic mapping metadata elements.

During the process of mapping, it is very useful to include additional attributes/columns in the SSSOM matrix in which information, upon which the mapping is based, can be stored.

We thus propose adding such columns during the process (e.g., definitions [*subject_definition*, *object_definition*], syntax requirements [*subject_valueSyntax*, *object_valueSyntax*]; see Approach: Mapping). Once the process is over, a leaner SSSOM product can be released omitting these supporting attributes.

For mapping keys from metadata standards to one another, this TG recommends:

1. Follow the SSSOM guidance*^32^.
2. Until official guidance is offered from the SSSOM team, apply the extension proposed above (see Approach: Mapping) to additionally capture the mapping of syntax requirements:
  1. using the SSSOM *predicate_id* and corresponding *comment* to capture the semantics, and the *syntax_predicate_id* and corresponding *syntax_comment* to capture the syntactic mapping of terms.
3. Communicate any needed extensions to the SSSOM team via their issue tracker*^33^.

#### Recommendations for many-to-one, many-to-many, one-to-many mappings

Due, in part, to the different approaches to atomization described above and below, many of the proposed relationships between MIxS and DwC keys required one-to-many or many-to-one mappings. This usually occurred when one specification offered multiple similar alternative keys for a phenomenon (e.g., DwC offers five keys relevant to “depth” measurements, while MIxS only offers one).

Recognizing that many keys in DwC or MIxS have community- and development-specific legacies, we recommend:

1. A mapping between metadata standards should be all-encompassing, and may thus include many-to-one, many-to-many, or one-to-many mappings.
2. Implementers, who represent a community of practice, can add notes on what keys they think are the most sensible.
3. In the long term, the standards agencies should aim to reduce the complexity of keys, moving towards atomization, to support more one-to-one relationships, eventually supporting full convergence.

#### Recommendations for semantic and syntactic alignment

DwC and MIxS specifications both offer guidance on the syntax expected for each value in a given key-value pair, alongside general notes on the expected semantics. In DwC, a value’s expected semantics*^34^ are captured in the *Definition* and *Notes* attributes of the List of Darwin Core Terms*^35^, while the *Examples* attribute shows expected value syntax. MIxS offers similar semantic guidance in the *Definition* attribute, with syntax and similar conventions specified in the *Expected value*, *Value syntax*, *Preferred unit*, and *Examples* attributes of the MIxS checklist*^36^. Since DwC and MIxS have been developed independently from one another, and complex/bespoke syntax is common to both specifications, there is considerable divergence in their conventions. These include:

- For measured values, MIxS expects the unit to be included as part of the value, while in DwC the unit is not allowed as part of the value - it is either inherent in the term definition or requires a separate term to specific the unit (optional for verbatim fields*^37^, which are expected to represent the measurement as originally recorded).
- For measured values, MIxS offers a “*preferred” unit* option, which - as the label implies - is not mandatory, while DwC either clearly defines the expected unit for a value or allows any unit to be used in a unit field related to the value field (except for verbatim fields).
- Some MIxS keys, such as lat_lon, expect values that capture two or more measured/derived values. DwC typically separates these measured/derived values across two or more keys (e.g., decimalLatitude and decimalLongitude).
- Also, several MIxS fields allow for a numeric value or a range, followed by a measurement unit (*size_frac*, *samp_size*, *temp*, *depth*, etc.). Darwin Core generally opts for atomic values associated with its keys.

Incompatibilities, such as those above, create (meta)data silos between communities using one or the other specification. Mappings built upon these can (in general) only be semantically and syntactically loose, and implementers must create and maintain converters or automated translators between the two, severely limiting and likely causing error propagation in machine-to-machine exchanges.

The SSSOM community is actively looking at ways to address these kinds of data structure mappings, and whether to address them as in scope for SSSOM, or to address these using the LinkML-transformer framework.

To secure improved semantic and syntactic alignment, this TG recommends the following:

1. The use of more explicit labels (terms), associated with less ambiguous definitions (many of which are more descriptive than definitional).
  1. Additionally, further cross-organization efforts to align the semantics of their fields in successive releases, using their obsolescence/change processes as appropriate.
2. Examples or descriptions of what is within and outside of the semantic scope/range of each field.
3. For any non-verbatim fields, clear guidance on what syntax is expected in each field (e.g., how many terms, separated how, with or without which unit?).
4. Re-use of existing and established terms from more general standards organizations within each specification (e.g., using dcterms:license to capture licensing information within MIxS and DwC).
5. Alignment to official external standards (e.g., using ISO 8601 to capture the time and date of an event)*^38^.
6. Synchronization between standards bodies ahead of new releases for closer syntactic alignment.
7. Semantic stability and standard syntax so stable converters can be written.
8. Atomic key-value structures, such that no complex or bespoke data structure exists in each value. For example, splitting ranges into dedicated start and stop fields.
  1. With advancement towards RDF- or JSON-based representations, allowing lists to be rendered as repeated key-value pairs.
9. Removing units from values by, for example, requiring a standard unit in the definition of each key.

#### Recommendations for the mapping of MIxS environmental package terms

In addition to MIxS core, MIxS contains numerous “environmental packages” which bundle keys which improve the contextualization of sequences in a given sampling environment. These are especially relevant for associating specific chemical and physical environmental measurements with specimens collected from these environments. Examples include marine, soil, food, and host-associated packages. These packages were created as a means to keep the core set relatively small, while rapidly accounting for the needs of sub-domains. These keys, and specifications of expected values, however, have not been harmonized or otherwise made interoperable with information standards published and used in Earth and environmental sciences.

Thus, this TG created SSSOM mappings and harmonization notes only for MIxS keys that directly pertained to sequences (MIxS core), rather than the specific environment they were obtained from.

Recognizing that the standardization domain/mandate of the GSC does not extend to standards of environmental parameters, this TG recommends that:

1. Any sustained reference implementation of a MIxS extension of DwC - endorsed by the GSC and TDWG - is limited to those MIxS keys which closely pertain to sequences (MIxS core), rather than the environments they originate from (MIxS environmental packages).
2. The GSC, as it begins to transition MIxS into RDF, should make efforts to map and eventually replace their environmental keys with equivalent, well-described keys from an information standards body working in the Earth and environment domain. We strongly advise that this is done as a joint activity with TDWG, to prevent decoupling and the need for downstream re-alignment.
3. Users wishing to use the MIxS environmental package keys in DwC Archives should use the MeasurementOrFact (MOF)*^39^ collection of keys (cast as an MoF class and associated properties, see Suppl. material 1 Section 2 for technical clarification) in the DwC specification. In our analysis, we found it valid to include a qualified mapping to a MIxS key URI as a value associated with the DwC “measurementRemark” key. This - alongside the other MOF key-value pairs - would allow any key in the MIxS environmental packages (either directly measured [measurement] or asserted to be true [fact]) to be represented in DwC. *^40^
  1. While we demonstrate how to link MIxS environmental package keys to DwC’s MoF, we draw attention to the fact that the GSC’s mandate is not within the standardization of Earth and environment metadata. Thus, where possible, users should attempt to use values from more Earth and environmental vocabularies, thesauri, ontologies, etc.
  2. Please see Suppl. material 1 Section 2 for an example of the above, and note the term measurementType.
4. TDWG and the GSC, in partnership with one or more standards bodies in the Earth and environmental sciences (e.g., the Earth Science Information Partners), convene a task group (or extend and expand this TG with a new mandate) to provide recommendations on how to sustainably and FAIRly incorporate well-adopted and more formally standardized environmental parameters into both MIxS and DwC.

To our knowledge, there is no sustained attempt to secure interoperability between the competing standards (most of which are informal, ad hoc, or de facto, as are MIxS and DwC) in this space. Some organizations and efforts of interest are listed below.

- Parameter vocabularies
  - The British Oceanographic Data Centre (BODC)*^41^ Natural Environment Research Council (NERC) Vocabularies*^42^, e.g., BODC Parameter Usage Vocabulary*^43^
- The Open Geospatial Consortium (OGC)*^44^
- Climate and Forecasting Variables*^45^

We note that, while this vacuum exists, implementers will create their own internal standards for expediency*^46^. This does provide some basis for later alignment but also creates overhead as more unaligned information standards are released, compete for users, and decouple information systems and communities. We, therefore, re-emphasize the need for both TDWG and the GSC to engage with information standards communities in the Earth and environment domain to integrate their specifications.

#### Recommendations for licensing information

Information on licensing is critical for data reusability (as declared in the FAIR Principles Wilkinson et al. 2016). Such information is captured in DwC through the import of the Dublin Core key dcterms:license*^47^; however, there is no equivalent key provided in the MIxS specification.

Recognizing that the GSC does not currently intend to extend their core checklist to include a key for licensing information*^48^, this TG recommends that implementers extend MIxS records with the Dublin Core key http://purl.org/dc/terms/license to capture data reuse restrictions.

While saying this, we also recognize the need for further discussions around the subject of license and reuse in conjunction with access and benefit sharing discussions around the Nagoya protocol and digital sequence information, as well as in conjunction with the implementation of the CARE principles Carroll et al. 2020 (e.g., through Traditional Knowledge and Biocultural Labels and Notices). Thus, additional and more nuanced fields expanding on reuse restrictions should be considered as they are being developed.

## Conclusion and outlook

In concluding this document, we emphasize the importance of convening a diverse and multi-stakeholder TG. With representatives from established biodiversity data infrastructures, domain experts, data generators, and publishers, we - ab initio - bridged the conceptual to the application space. We leveraged this to 1) generate, and internally review, a fine-grained mapping in a standard format, 2) implement new extensions to DwC, and 3) develop recommendations on how to expand on and sustain these. We have also identified areas of concern, which are in need of further attention and follow-up TGs.

Despite the achievements above, the work of this TG falls short of making an automated conversion possible. For this to be achievable, both community standards require further semantic and syntactic alignment, both between one another and with external data-on-the-web standards and best practices. In general, avoiding bespoke value syntax and complex semantics associated with keys (e.g., by unpacking complex keys into a number of simpler ones) will help this effort.

As stated in our MoU, the sustainability of this TG’s output must be ensured through aligned processes within the community standards bodies involved. As noted in Suppl. material 1 Section 3, we recommend that the sustainability of this TG’s outputs are further secured, and protected from ad-hoc changes, by creating a follow-up TG to develop a MIxS-driven vocabulary enhancement*^49^ based on the MIxS-DwC extension. All of this is working towards a state where, as soon as an updated specification is released, the possibility of automatic data translation between standards exists and is validated.

In the long term, as sequence-based (meta)data becomes more central to biodiversity observing, we anticipate a full convergence of these standards. Simultaneously, tools to converge records built from these specifications into more machine-readable forms (e.g., RDF triples), would increase their value, scalability, and portability.

We trust that the activities of this TG will inspire similar activities between other metadata standards in this space, to break down silos and open a path to a more collaborative and interoperable future.

## Re-use potential

Our approach demonstrates considerable reuse potential, with comprehensive documentation of each step for easy adoption and adaptation by others. In Suppl. material 1 Section 3, in addition to the recommendations in the main manuscript, we provide further recommendations for future TGs.

We see our TG’s diverse membership and perspectives as a strong model to follow in future work developing or interlinking community standard specifications used by many stakeholders. Through this, operational realities, technical soundness, and policy-level perspectives can be better integrated and built upon. Further, leveraging pre-existing and standardized resources such as SSSOM and SKOS has streamlined the process and allows the mapping to be easily and broadly parsed and understood.

We encourage others to consider and further develop our approach. This will happen in the DwC/MIxS world, as new versions are released of each standard and the mapping, but it has also already started with other (meta)data standards, such as GGBN*^50^; recognizing the value added by systematically and authoritatively mapping and interlinking converging (meta)data standards. The establishment of a Standards Mapping TG under the TDWG Technical Architecture Group, which is currently underway, reinforces the importance of this work and will provide a platform for furthering guidelines for developing and documenting mappings.

We envision this as a significant step toward fostering a collaborative digital ecosystem, where reduced redundancy and increased interoperability become the norm.

## Data resources

The described TG outputs (V2.1.0) are hosted in the "dwc-mixs" folder of GBWG GitHub directory. The permanent identifier to this repository and its content is https://doi.org/10.5281/zenodo.8393224.

- Mappings
- Extension
- MoU

The versions of the standards specifications used can be found:

- here for DwC (Version 2021-03-29; most current official version as of the development of TG outputs)
- here for MIxS (Version 5; most current official version as of the development of TG outputs)

The TG discussions can be found on the GBWG issue tracker with the label "DwC-MIxS TG". We encourage potential users to contribute to those discussions and/or request improvements as needed. For feedback on the V2.1.0 release specifically, please use the "DwC-MIxS feedback V2.1.0" label.

## Supplementary Material

A3FAC521-5598-5956-86B5-771A3CD6323F Supplementary material 1 Aligning Standards Communities for Omics Biodiversity Data: Sustainable Darwin Core-MIxS Interoperability - Appendices Data type Text Brief description Includes Sections on (1) Exemplar rows from the SSSOM mapping files, (2) Using MIxS environmental package keys in DwC Archives, (3) Issues noted for future TGs, (4) Relation of interoperable standards to the future of data-driven publishing File: oo_915176.docx https://binary.pensoft.net/file/915176 As in manuscript

## Figures and Tables

**Table 1.** Glossary

Term	Definition
Darwin Core (DwC)	A specification released by TDWG that includes a glossary of terms intended to facilitate the sharing of information about biological diversity by providing identifiers, labels, and definitions (in this document, unless otherwise specified, we refer to DwC Version 2021-03-29) *^3^
Darwin Core Archive (DwC-A)	A dataset that 1) contains data about species occurrences, checklists, sampling events and/or material sample data and 2) makes use of Darwin Core terms to qualify fields. DwC-A records comprise a set of text (CSV) files with a simple descriptor record (i.e. meta.xml) to inform others how your files are organized. The format is defined in the Darwin Core Text Guidelines. It is the preferred format for publishing data to the GBIF and OBIS networks.
Darwin Core Extension	A list of defined keys to be used in combination with/in addition to DwC keys to create a more complete metadata record for a given situation. *^4^
Minimum Information about any (x) Sequence (MIxS)	A collection of checklists released by the GSC to define both the minimal and extended metadata associated with any sequencing record (in this document, unless otherwise specified, we refer to MIxS Version 5). *^5^
MIxS core	A MIxS checklist providing minimal (and extended) sets of metadata keys directly related to the sequences.
MIxS environmental packages	A collection of MIxS checklists providing extended sets of metadata keys about different sampling environments, deemed important by the MIxS user community.
Simple Knowledge Organization System Reference (SKOS)	A common data model for sharing and linking knowledge organization systems via the Web. It provides a lightweight, intuitive language for developing and sharing new knowledge organization systems.
Simple Standard for Sharing Ontology Mappings (SSSOM)	A catalog of minimal and standard metadata elements for the dissemination of mappings between ontology terms.

**Table 2.** **Table 2**: Metadata elements additionally added to the DwC-MIxS mapping document to capture the syntactic mapping between keys. Please see an example of how these keys were used in the mapping in the Suppl. material 1 Section 1.

Element ID	Description	TSV/RDF Example
syntax_predicate_id	The ID of the predicate or relation that relates the syntax of the subject and object of this match.	skos:relatedMatch
syntax_comment	Free text field containing either curator notes or text generated by a tool providing additional informative information on the syntactic mapping.	The subject expects a verbatim input (so anything really), while the object expects a {float} {unit} entry.

**Table 3.** **Table 3**: Metadata elements additionally added to the working document for the SSSOM mapping between DwC and MIxS keys. These metadata elements were additionally added to facilitate the mapping process by having all the information needed as part of one spreadsheet. Please see an example of how these keys were used in the mapping in Suppl. material 1 Section 1.

Element ID	Description	TSV/RDF Example
subject_definition	The definition of the subject of this mapping.	The original description of the depth below the local surface.
subject_valueSyntax	The value syntax expected for the subject of this mapping.	verbatim
syntax_predicate_id	The ID of the predicate or relation that relates the syntax of the subject and object of this match.	skos:relatedMatch
syntax_predicate_label	The label of the predicate/relation of the syntactic mapping.	related match to
object_definition	The definition of the object of this mapping.	Depth is defined as the vertical distance below local surface, e.g., for sediment or soil samples depth is measured from sediment or soil surface, respectively. Depth can be reported as an interval for subsurface samples.
object_valueSyntax	The value syntax expected for the object of this mapping.	{float} {unit}
syntax_comment	Free text field containing either curator notes or text generated by a tool providing additional informative information on the syntactic mapping.	The subject expects a verbatim input (so anything really), while the object expects a {float} {unit} entry.

## Footnotes

*1 This example assumes that the corresponding unit of the value is defined in the metadata associated with the key. See Recommendations for semantic and syntactic alignment.

*2 In the proceedings of this TG, it was noted that the loose usage of such terms referencing the linguistic artifacts (e.g., “terms”) and the more technical data structures (“key-value pairs”) can produce confusion during tasks that require semantic precision, including this mapping. Thus our clarification here.

*3
http://rs.tdwg.org/dwc/doc/list/2021-03-29

*4
https://rs.gbif.org/extension/

*5
https://github.com/GenomicsStandardsConsortium/mixs/tree/MIxS5

*6 Currently the SSSOM community is working to provide best practice for these situations; see https://github.com/tdwg/gbwg/issues/54, https://github.com/mapping-commons/SSSOM/issues/52, https://github.com/mapping-commons/SSSOM/issues/56.

*7 For example, one of the challenges with mapping different term lists is that frequently we see that one system bakes in a unit to the meaning of the term, and the other system has a corresponding term whose value expects a compound of value plus unit.

*8 https://github.com/tdwg/gbwg/tree/main/dwc-mixs. An exemplar row from the mapping files can additionally be found in the Suppl. material 1 Section 1.

*9 ORCID: 0000-0002-6601-2165

*10 ORCID: 0000-0002-2411-565X

*11
http://rs.gbif.org/schema/extension.xsd

*12
http://rs.gbif.org/schema/thesaurus.xsd

*13
https://www.ebi.ac.uk/metagenomics/

*14 GBIF: See some examples here https://www.gbif.org/dataset/9e29a2fe-d780-48a8-a93f-9ce041f9202f, https://www.gbif.org/dataset/9f0e1ca6-fb08-4c72-9a4a-1e3b7a528c10, https://www.gbif.org/dataset/4cefd38b-8ada-46e0-9ef7-3531f8a204df, https://www.gbif.org/dataset/9d7baaac-57db-4852-9993-7f0e7f15635b OBIS: See some examples at https://obis.org/datasets, select "DNADerivedData" as data type

*15
https://github.com/tdwg/dwc/labels/Task%20Group%20-%20Material%20Sample

*16
https://github.com/tdwg/gbwg/tree/v2.1.0/dwc-mixs/mapping

*17
https://docs.google.com/spreadsheets/d/1QDeeUcDqXes69Y2RjU2aWgOpCVWo5OVsBX9MKmMqi_o/edit#gid=0

*18 https://github.com/tdwg/gbwg/tree/v2.1.0/dwc-mixs/mapping. An exemplar row from the mapping files can additionally be found in the Suppl. material 1 Section 1.

*19
https://github.com/tdwg/gbwg/blob/v2.1.0/dwc-mixs/mapping/DwC-MIxS_mappingSemantic.sssom.tsv

*20
https://github.com/tdwg/gbwg/blob/v2.1.0/dwc-mixs/mapping/DwC-MIxS_mappingSyntactic.sssom.tsv

*21
https://github.com/tdwg/gbwg/blob/v2.1.0/dwc-mixs/mapping/DwC-MIxS_mappingSupport.sssom.tsv

*22
https://github.com/GenomicsStandardsConsortium/mixs/issues

*23
https://github.com/tdwg/dwc/issues

*24
https://github.com/tdwg/gbwg/tree/v2.1.0/dwc-mixs/dwc

*25
https://github.com/tdwg/gbwg/blob/v2.1.0/dwc-mixs/dwc/extension/mixs_darwin_core_extension.xml

*26
https://github.com/tdwg/gbwg/tree/v2.1.0/dwc-mixs/dwc#mixs-terms-excluded-from-the-extension

*27
https://github.com/tdwg/gbwg/blob/v2.1.0/dwc-mixs/dwc/extension/mixs_darwin_core_extension.xml

*28 See the meta.xml file of the Korean Peninsula Flora as an example of how an XML file is used as part of the DwC-A: https://www.gbif.org/dataset/e09e1e1f-2460-4017-a964-e999abd2bf66

*29
https://rs.gbif.org/extension/gbif/1.0/dna_derived_data_2021-07-05.xml

*30
https://github.com/GenomicsStandardsConsortium/mixs/issues

*31
https://github.com/tdwg/dwc/issues

*32
https://mapping-commons.github.io/sssom/

*33
https://github.com/mapping-commons/SSSOM/issues

*34 In both MIxS and DwC, multiple definitions suffer from ambiguity, circularity, or other semantic aberrations. An effort to improve these would also improve future mapping and (meta)data (re)use efforts.

*35
http://rs.tdwg.org/dwc/doc/list/​​​​​​​

*36
https://gensc.org/mixs/

*37 Verbatim fields are essential to collect specimen data from museums, etc.

*38 The rare occasion where DwC and MIxS semantically and syntactically matched exactly was due to external standards (ISO 8601)

*39
https://dwc.tdwg.org/terms/#measurementorfact

*40 Please see Suppl. material 1 Section 1 for an example of this.

*41
https://www.bodc.ac.uk

*42
http://vocab.nerc.ac.uk

*43
http://vocab.nerc.ac.uk/collection/P01/current/

*44
https://www.ogc.org/

*45
https://www.w3.org/2005/Incubator/ssn/ssnx/cf/cf-property

*46 For example, GBIF is building basic vocabularies in SKOS, based on the values they see in the aggregation of original sources. The objective here is more to clean data than to build rigorous vocabularies. Such internal efforts would greatly benefit from having a consolidated, appropriately endorsed, and standardised specification of environmental terms to align to.

*47 License information is additionally captured on the dataset level in a DwC-A in EML, however, this declaration may not carry through automatically to the record in the dataset.

*48
https://github.com/GenomicsStandardsConsortium/mixs/issues/111#issuecomment-790759090

*49 Similar to the Chronometric Age vocabulary enhancement https://tdwg.github.io/chrono/terms/

*50 See https://github.com/tdwg/gbwg/issues?q=is%3Aopen+is%3Aissue+label%3A%22GGBN-MIxS+Mapping%22

## References

[B10525949] Carroll Stephanie Russo, Garba Ibrahim, Figueroa-Rodríguez Oscar L., Holbrook Jarita, Lovett Raymond, Materechera Simeon, Parsons Mark, Raseroka Kay, Rodriguez-Lonebear Desi, Rowe Robyn, Sara Rodrigo, Walker Jennifer D., Anderson Jane, Hudson Maui (2020). The CARE Principles for Indigenous Data Governance. Data Science Journal.

[B9957147] Field Dawn, Amaral-Zettler Linda, Cochrane Guy, Cole James R., Dawyndt Peter, Garrity George M., Gilbert Jack, Glöckner Frank Oliver, Hirschman Lynette, Karsch-Mizrachi Ilene, Klenk Hans-Peter, Knight Rob, Kottmann Renzo, Kyrpides Nikos, Meyer Folker, San Gil Inigo, Sansone Susanna-Assunta, Schriml Lynn M., Sterk Peter, Tatusova Tatiana, Ussery David W., White Owen, Wooley John (2011). The Genomic Standards Consortium. PLoS Biology.

[B10081857] Franco Diego C., Signori Camila N., Duarte Rubens T. D., Nakayama Cristina R., Campos Lúcia S., Pellizari Vivian H. (2017). High prevalence of Gammaproteobacteria in the sediments of Admiralty Bay and North Bransfield Basin, Northwestern Antarctic Peninsula. Frontiers in Microbiology.

[B10081905] Hu Yue O. O., Karlson Bengt, Charvet Sophie, Andersson Anders F. (2016). Diversity of pico- to mesoplankton along the 2000 km salinity gradient of the Baltic Sea. Frontiers in Microbiology.

[B10080806] Matentzoglu Nicolas, Balhoff James P, Bello Susan M, Bizon Chris, Brush Matthew, Callahan Tiffany J, Chute Christopher G, Duncan William D, Evelo Chris T, Gabriel Davera, Graybeal John, Gray Alasdair, Gyori Benjamin M, Haendel Melissa, Harmse Henriette, Harris Nomi L, Harrow Ian, Hegde Harshad B, Hoyt Amelia L, Hoyt Charles T, Jiao Dazhi, Jiménez-Ruiz Ernesto, Jupp Simon, Kim Hyeongsik, Koehler Sebastian, Liener Thomas, Long Qinqin, Malone James, McLaughlin James A, McMurry Julie A, Moxon Sierra, Munoz-Torres Monica C, Osumi-Sutherland David, Overton James A, Peters Bjoern, Putman Tim, Queralt-Rosinach Núria, Shefchek Kent, Solbrig Harold, Thessen Anne, Tudorache Tania, Vasilevsky Nicole, Wagner Alex H, Mungall Christopher J (2022). A simple standard for sharing ontological mappings (SSSOM). Database.

[B10081914] Svenningsen Cecilie S., Frøslev Tobias Guldberg, Bladt Jesper, Pedersen Lene Bruhn, Larsen Jonas Colling, Ejrnæs Rasmus, Fløjgaard Camilla, Hansen Anders Johannes, Heilmann-Clausen Jacob, Dunn Robert R., Tøttrup Anders P. (2021). Detecting flying insects using car nets and DNA metabarcoding. Biology Letters.

[B10080654] Tuama Éamonn Ó, Deck John, Dröge Gabriel, Döring Markus, Field Dawn, Kottmann Renzo, Ma Juncai, Mori Hiroshi, Morrison Norman, Sterk Peter, Sugawara Hideaki, Wieczorek John, Wu Linhuan, Yilmaz Pelin (2012). Meeting Report: Hackathon-Workshop on Darwin Core and MIxS standards alignment (February 2012). Standards in Genomic Sciences.

[B10080798] UNESCO-IOC (2021). The United Nations decade of ocean science for sustainable development (2021-2030) Implementation Plan. https://unesdoc.unesco.org/ark:/48223/pf0000377082.locale=en.

[B10080641] Wieczorek John, Bloom David, Guralnick Robert, Blum Stan, Döring Markus, Giovanni Renato, Robertson Tim, Vieglais David (2012). Darwin Core: An evolving community-developed biodiversity data standard. PLoS ONE.

[B10260077] Wilkinson Mark D., Dumontier Michel, Aalbersberg IJsbrand Jan, Appleton Gabrielle, Axton Myles, Baak Arie, Blomberg Niklas, Boiten Jan-Willem, da Silva Santos Luiz Bonino, Bourne Philip E., Bouwman Jildau, Brookes Anthony J., Clark Tim, Crosas Mercè, Dillo Ingrid, Dumon Olivier, Edmunds Scott, Evelo Chris T., Finkers Richard, Gonzalez-Beltran Alejandra, Gray Alasdair J. G., Groth Paul, Goble Carole, Grethe Jeffrey S., Heringa Jaap, ’t Hoen Peter A. C, Hooft Rob, Kuhn Tobias, Kok Ruben, Kok Joost, Lusher Scott J., Martone Maryann E., Mons Albert, Packer Abel L., Persson Bengt, Rocca-Serra Philippe, Roos Marco, van Schaik Rene, Sansone Susanna-Assunta, Schultes Erik, Sengstag Thierry, Slater Ted, Strawn George, Swertz Morris A., Thompson Mark, van der Lei Johan, van Mulligen Erik, Velterop Jan, Waagmeester Andra, Wittenburg Peter, Wolstencroft Katherine, Zhao Jun, Mons Barend (2016). The FAIR Guiding Principles for scientific data management and stewardship. Scientific Data.

[B10080538] Yilmaz Pelin, Kottmann Renzo, Field Dawn, Knight Rob, Cole James R, Amaral-Zettler Linda, Gilbert Jack A, Karsch-Mizrachi Ilene, Johnston Anjanette, Cochrane Guy, Vaughan Robert, Hunter Christopher, Park Joonhong, Morrison Norman, Rocca-Serra Philippe, Sterk Peter, Arumugam Manimozhiyan, Bailey Mark, Baumgartner Laura, Birren Bruce W, Blaser Martin J, Bonazzi Vivien, Booth Tim, Bork Peer, Bushman Frederic D, Buttigieg Pier Luigi, Chain Patrick S G, Charlson Emily, Costello Elizabeth K, Huot-Creasy Heather, Dawyndt Peter, DeSantis Todd, Fierer Noah, Fuhrman Jed A, Gallery Rachel E, Gevers Dirk, Gibbs Richard A, Gil Inigo San, Gonzalez Antonio, Gordon Jeffrey I, Guralnick Robert, Hankeln Wolfgang, Highlander Sarah, Hugenholtz Philip, Jansson Janet, Kau Andrew L, Kelley Scott T, Kennedy Jerry, Knights Dan, Koren Omry, Kuczynski Justin, Kyrpides Nikos, Larsen Robert, Lauber Christian L, Legg Teresa, Ley Ruth E, Lozupone Catherine A, Ludwig Wolfgang, Lyons Donna, Maguire Eamonn, Methé Barbara A, Meyer Folker, Muegge Brian, Nakielny Sara, Nelson Karen E, Nemergut Diana, Neufeld Josh D, Newbold Lindsay K, Oliver Anna E, Pace Norman R, Palanisamy Giriprakash, Peplies Jörg, Petrosino Joseph, Proctor Lita, Pruesse Elmar, Quast Christian, Raes Jeroen, Ratnasingham Sujeevan, Ravel Jacques, Relman David A, Assunta-Sansone Susanna, Schloss Patrick D, Schriml Lynn, Sinha Rohini, Smith Michelle I, Sodergren Erica, Spor Aymé, Stombaugh Jesse, Tiedje James M, Ward Doyle V, Weinstock George M, Wendel Doug, White Owen, Whiteley Andrew, Wilke Andreas, Wortman Jennifer R, Yatsunenko Tanya, Glöckner Frank Oliver (2011). Minimum information about a marker gene sequence (MIMARKS) and minimum information about any (x) sequence (MIxS) specifications. Nature Biotechnology.

