# Supplementary material for: Aligning Standards Communities for Omics Biodiversity Data: Sustainable Darwin Core-MIxS Interoperability
Source: Biodivers Data J. 2023 Oct 3;11:e112420. doi: 10.3897/BDJ.11.e112420 (PMC10565567; doi:10.3897/BDJ.11.e112420)
Supplement: Supplementary material 1 — Aligning Standards Communities for Omics Biodiversity Data: Sustainable Darwin Core-MIxS Interoperability - Appendices [file bdj-11-e112420-s001.docx]

# Aligning Standards Communities for Omics Biodiversity Data: Sustainable Darwin Core-MIxS Interoperability - Appendices

# Appendix 1

## Exemplar rows from the SSSOM mapping files

Presented here are exemplar rows from each of the three SSSOM mapping files presented in this paper. The highlighting of the exemplar rows aims to facilitate readers' comprehension of the mapping structure without necessitating a thorough review of the entire mapping files accessible on GitHub.

Consistent with the examples used in Tables 2 and 3 in the main manuscript text, we are using the mapping of dwc:verbatimDepth to MIXS:0000018 as an example.

For the sake of readability in this Supplementary Material section, we have transposed the table content (meaning what you see here as rows are columns in the original mapping files and vice versa).

**Table 1** **Exemplar rows from the SSSOM mapping files (Semantic, Syntactic, Support).**

In cases, in which a key is not associated with a value, the field will remain blank (e.g., definitions are exclusively present in the Support mapping file, thus any definition fields for the Semantic or Syntactic mapping will remain blank in this table).

| **Key** | **Value in Semantic SSSOM mapping file** | **Value in Syntactic SSSOM mapping file** | **Value in Support SSSOM mapping file** |
| --- | --- | --- | --- |
| subject_id | dwc:verbatimDepth | dwc:verbatimDepth | dwc:verbatimDepth |
| subject_label | Verbatim Depth | Verbatim Depth | Verbatim Depth |
| subject_definition |  |  | The original description of the depth below the local surface. |
| subject_valueSyntax |  |  | verbatim |
| subject_category | https://dwc.tdwg.org/list/#dcterms_Location | https://dwc.tdwg.org/list/#dcterms_Location | https://dwc.tdwg.org/list/#dcterms_Location |
| predicate_id | skos:exactMatch |  | skos:exactMatch |
| predicate_label |  |  | has exact match |
| syntax_predicate_id |  | skos:relatedMatch | skos:relatedMatch |
| syntax_predicate_label |  |  | has related match |
| object_id | MIXS:0000018 | MIXS:0000018 | MIXS:0000018 |
| object_label | depth | depth | depth |
| object_definition |  |  | Please refer to the definitions of depth in the environmental packages |
| object_valueSyntax |  |  | - |
| object_category | MIxS core | MIxS core | MIxS core |
| match_type | HumanCurated | HumanCurated | HumanCurated |
| creator_id | orcid:0000-0003-1144-0290 \| orcid:0000-0002-2996-719X |  | orcid:0000-0003-1144-0290 \| orcid:0000-0002-2996-719X |
| syntax_creator_id |  | orcid:0000-0003-1144-0290 | orcid:0000-0003-1144-0290 |
| subject_source | dwc:verbatimDepth | dwc:verbatimDepth | dwc:verbatimDepth |
| subject_source_version | dwcversion:verbatimDepth-2017-10-06 | dwcversion:verbatimDepth-2017-10-06 | dwcversion:verbatimDepth-2017-10-06 |
| object_source | MIXSversion:mixs_v5.xlsx | MIXSversion:mixs_v5.xlsx | MIXSversion:mixs_v5.xlsx |
| object_source_version | 5 | 5 | 5 |
| mapping_date | 08.04.22 |  | 08.04.22 |
| syntax_mapping_date |  | 16.04.21 | 16.04.21 |
| confidence | 1 |  | 1 |
| syntax_confidence |  | 1 | 1 |
| comment | Both terms focus on a depth/vertical distance *below* some surface. Note that in Darwin Core, according to best practices, depth is measured in water from an air-water surface, while in other media the corresponding terms are dwc:minimumDistanceAboveSurfaceInMeters and dwc:maximumDistanceAboveSurfaceInMeters, where the values would be negative for depth. Thus, for a sediment at the bottom of a lake, there would be an elevation of the lake surface, the depth of the top of the sediment in the lake, and minimum and maximum distances below the sediment surface within which a sample was taken. |  | Both terms focus on a depth/vertical distance *below* some surface. Note that in Darwin Core, according to best practices, depth is measured in water from an air-water surface, while in other media the corresponding terms are dwc:minimumDistanceAboveSurfaceInMeters and dwc:maximumDistanceAboveSurfaceInMeters, where the values would be negative for depth. Thus, for a sediment at the bottom of a lake, there would be an elevation of the lake surface, the depth of the top of the sediment in the lake, and minimum and maximum distances below the sediment surface within which a sample was taken. |
| syntax_comment |  | The DwC term expects a verbatim input (so anything really), while MIxS expects a {float} {unit} entry in meters. Any verbatim terms in DwC are matched with relatedMatch to more prescribed terms in MIxS, as they can have free text (or any other string-based value) as syntax. | The DwC term expects a verbatim input (so anything really), while MIxS expects a {float} {unit} entry in meters. Any verbatim terms in DwC are matched with relatedMatch to more prescribed terms in MIxS, as they can have free text (or any other string-based value) as syntax. |

.

# Appendix 2

## Using MIxS environmental package keys in DwC Archives

To illustrate the Recommendation for the mapping of MIxS environmental package terms, we have added an example for moving from a MIxS-compliant temperature measurement to a DwC-compliant temperature record (see Table 3 and the RDF serialization below).

The simple temperature key-value pair from MIxS offers information on the following DwC properties from the MoF class: dwc:MeasurementOrFact, dwc:measurementType, dwc:measurementValue, dwc:measurementUnit (see Table 3). However, the MIxS specification currently falls short from providing fields to capture the, not less relevant, information on the measurement method [dwc:measurementMethod], the person taking the measurement [dwc:measurementDeterminedBy], and the accuracy in the measurement [measurementAccuracy], or any other remarks [dwc:measurementRemarks]. While there are some MIxS fields to capture e.g. the method used for a certain procedure (e.g. MIXS:0000002 samp_collect_device, which expects the method or device employed for collecting the sample type), there are no such options for any of the environmental package fields.

Especially anticipating the broader use of Essential Ocean Variables (EOVs) from the Global Ocean Observing System (GOOS), we will need to find a coherent and consistent way of reporting not only on the value of a certain measurement, but also the method.^^[[1]](#footnote-1)^^ To accommodate this need identified by the community, sustainable solutions will need to be developed and implemented to capture the exact method used to obtain a measurement value also in MIxS-compliant records (e.g. through an extension of the MIxS specification, or by using a combination of MIxS and the DwC MoF extension, ...).

**Table 1: Translation of MIxS environmental package key-value pairs into DwC MeasurementOrFact key-value pairs.** Prefix expansions: 1) “mixs:” = “https://w3id.org/gensc/terms/” 2) “dwc:” = “http://rs.tdwg.org/dwc/terms/”

| **MIxS key-value pair** | | **DwC key-value pairs** | |
| --- | --- | --- | --- |
| mixs:MIXS:0000742 | 17 °C | dwc:MeasurementOrFact | Temperature in Celsius of the sample at the time of sampling |
|  |  | dwc:measurementType | temperature |
|  |  | dwc:measurementValue | 17 |
|  |  | dwc:measurementUnit | °C |
|  |  | dwc:measurementRemark | The values of dwc:measurementValue and dwc:measurementUnit captured here are a near match to the expected value of MIXS:0000742, which combines the two in a string |

### Exemplar RDF to express MIxS environmental keys as DwC Measurement or Fact triples

Below, we have translated the information captured in Table 3 into RDF. We are using very expanded RDF to make sure that we are as unambiguous as possible. **Please note that this is a draft, and that the MIxS IRIs may still be subject to change, as they have not yet been officially released.**

PREFIX : <http://example.dwc-mixs.org/>

PREFIX rdf: <http://www.w3.org/1999/02/22-rdf-syntax-ns#>

PREFIX rdfs: http://www.w3.org/2000/01/rdf-schema#

PREFIX xsd: <http://www.w3.org/2001/XMLSchema#>

PREFIX mixs: <https://w3id.org/gensc/terms/>

PREFIX dwc: <http://rs.tdwg.org/dwc/terms/>

PREFIX dwcA: <http://rs.tdwg.org/dwc/terms/attributes/>

# OUR_IRI is an IRI that dereferences to a value in a data table.

# MIxS is currently castings its keys as owl:ObjectProperties,

# see <https://github.com/cmungall/mixs-source> for up-to-date

# information.

mixs:MIXS:0000742 rdf:type rdf:Property .

mixs:MIXS:0000742 rdfs:range xsd:string .

# the value of the MIxS property must be string

# because of unit splicing, see comments on atomicity above

OUR_IRI mixs:MIXS:0000742 "17 °C" .

# We now define some DwC MoF classes and properties

dwc:MeasurementOrFact rdf:type rdfs:Class .

dwc:measurementValue rdf:type rdf:Property .

dwc:measurementUnit rdf:type rdf:Property .

dwc:measurementType rdf:type rdf:Property .

dwc:measurementRemarks rdf:type rdf:Property .

# We use a property from DwC attributes to link these

# properties to the MoF class. See the following for documentation:

# <https://github.com/tdwg/rdf/blob/master/Beginners4Vocabularies.md#461-informational-properties>

# In terms of RDF, this is not strictly necessary, but reflects

# the internal organisation of the DwC specification which may

# ease discovery of which properties to use when using a DwC

# class

dwc:measurementValue dwcA:organizedInClass dwc:MeasurementOrFact .

dwc:measurementUnit dwcA:organizedInClass dwc:MeasurementOrFact .

dwc:measurementType dwcA:organizedInClass dwc:MeasurementOrFact .

dwc:measurementRemarks dwcA:organizedInClass dwc:MeasurementOrFact .

# We now map the MIxS key to the DwC key with a skos

# predicate, loosely.
# Because the syntax and content of the MIxS value is

# non-atomic and the MoF splits units and the quantities

# they qualify, we can’t state stronger equivalence.

# Here, we state that the MIXS:0000742 key is narrower

# in conceptual scope than dwc:measurementType

dwc:measurementType skos:narrowMatch mixs:MIXS:0000742 .

# State that OUR_IRI is an instance of dwc:MeasurementOrFact

OUR_IRI rdf:type dwc:MeasurementOrFact .

# As the MoF class expects a description as a value, state what the data is about using the MIxS definition

OUR_IRI rdf:value "Temperature of the sample at the time of sampling"^^xsd:string .

# We add some remarks to explain the measures taken to

# cast the MIxS field to DwC

OUR_IRI dwc:measurementRemarks "The values of measurementValue and measurementUnit captured here are a near match to the expected value of https://w3id.org/gensc/terms/MIXS:0000742, which combines the two in a string" .

# Now add values using our DwC MoF properties as predicates

# Note that we define the datatypes in line as these may

# vary across MIxS keys

# We use an IRI for temperature from PATO for machine-readability

OUR_IRI dwc:measurementType http://purl.obolibrary.org/obo/PATO_0000146 .

# If we had a more specific type of temperature

# measurement , e.g. sea surface temperature, we could

# (and should) use an IRI with more specific semantics

#
# OUR_IRI dwc:measurementType http://purl.obolibrary.org/obo/ENVO_04000002 .

# We now set the remaining properties using literals

OUR_IRI dwc:measurementValue 17^^xsd:decimal .

OUR_IRI dwc:measurementUnit "°C"^^xsd:string .

#

#

# Appendix 3

## Issues noted for future TGs

The following issues were noted during the proceedings of this TG, which require the convening of subsequent TGs with appropriate scope.

### MIxS-driven vocabulary enhancement

The development of a MIxS-driven vocabulary enhancement to TDWG/DwC would provide additional protection from ad-hoc changes and improve the sustainability of this TG’s outputs by bringing them into the official TDWG standards space. A TG aiming to create a such a vocabulary enhancement could follow the example of the Chronometric Age vocabulary enhancement^^[[2]](#footnote-2)^^.

The development of a MIxS-driven vocabulary enhancement to TDWG/DwC would entail the following steps:

1. Based on this TG’s MIxS-DwC extension, create a csv file that contains the complete descriptions and definitions of the MIxS core keys that cannot be mapped to DwC and that are currently part of the MIxS-DwC extension; re-using the MIxS IRIs to identify the MIxS keys.
2. Use existing scripts to produce a quick reference guide from 1) and a term list document containing normative content.
3. Follow the Vocabulary Maintenance Standard specification for a public review of the vocabulary enhancement.
   1. As this is part of aligning two standards bodies - with the proposed terms being pre-defined and governed by a different standards body - the review process would be limited to the overall concept of the extension and usage comments and examples noted as part of DwC.
   2. Requested changes regarding the term definitions and descriptions could be suggested to the GSC directly via the MIxS issue tracker and could be considered for future MIxS versions.
   3. Precedent: Adoption of existing Dublin Core terms in DwC, for which the definitions are out of the TDWG jurisdiction, but for which the usage comments about how to use it with Darwin Core and the examples are open for change.
4. Incorporate review comments.
5. Move the vocabulary enhancement into operation.

### Controlled vocabularies in DwC to promote improved consistency and DwC-MIxS alignment / Improved semantic control through term lists from a curated list of ontologies

The general benefits of using open, sustained, community-driven, and quality controlled vocabularies, thesauri, or ontologies aligned to the FAIR Principles are many. In the context of this TG, doing so would greatly enhance the stability of semantic and syntactic mappings between keys and values, as well as conversions between them.

Previous efforts to incorporate FAIR terminological resources have been pursued by both communities, e.g. [recommendations to use the Environment Ontology (ENVO) or ontologies interoperating with it](https://github.com/EnvironmentOntology/envo/wiki/Using-ENVO-with-MIxS) in the mandatory elements of a MIxS-compliant record. In this case, interoperation potential is increasing as DwC considers similar content for the dwc:biome key (see below). There are other keys where agreeing upon a relatively small controlled vocabulary (either extant or yet-to-be-developed) for the values of a given key-value pair would be quite straightforward.

The following observations and considerations are offered to TG conveners who wish to take this issue forward:

1. As a result of the Darwin Core Public Review concluded on 2021-05-31, the recommendation was to commit [the issue proposing a new key 'dwc:biome'](https://github.com/tdwg/dwc/issues/38) and its dwciri: analog to a task group. Though there was general agreement on the utility of the new key for Darwin Core, there were several concerns raised about using a community ontology like ENVO directly. These included that the syntax used to add terms from ENVO (or other ontologies) to MIxS (e.g. *tropical moist broadleaf forest biome* [ENVO:01000228]) was not an approach which the DwC community currently uses, and concerns were raised about the chance of input errors arising. An alternative proposed by Steve Baskauf involves 1) The creation of a local, DwC controlled vocabulary with terms following patterns that have been adopted for other Darwin Core terms (e.g., dwc:establishmentMeans), 2) Linking these terms to OBO ontologies for the purpose of interoperability and definition.
2. The [new key proposal for dwc:environmentalMaterial](https://github.com/tdwg/dwc/issues/40) was not included in the recent Darwin Core Public Review as there was insufficient demand demonstrated via the DwC proposal process. With support from the MIxS stakeholders, this proposal could be promoted for inclusion in the TG described above for dwc:biome, as it would be of service in aligning the two specifications.
3. Though not included in the outputs of this task group, controlled vocabularies for a selection of mapped MIxS key-value pairs should also be created and socialised by both TDWG and the GSC.
   The recommendation from this group is to create these controlled vocabularies as a vocabulary enhancement set under a new task group. Part of the TG’s mission will be to include a way for the vocabularies associated with each key to be accessible to both MIxS users (e.g. submitting to the INSDC) and DwC users (submitting to OBIS or GBIF)
   Potential technical and operational issues were identified in how these vocabularies should be encoded and distributed. For example:
   - In order to include the terms from controlled vocabularies in software such as the Integrated Publishing Toolkit (IPT), “dummy” extension to a key, IRIs are created, such as
     dc:URI='https://rs.gbif.org/vocab/dna/decontam_software/anvi_o'. To minimise confusion, it was decided to use the GBIF namespace for this.
   - In the above, “/contigs” is one of the values expected in a controlled vocabulary for [https://w3id.org/gensc/terms/MIXS:0000005](https://w3id.org/gensc/terms/MIXS:0000005/contigs)
   - However, the IRI with the dummy suffix is not maintained or endorsed by the GSC, risking decoupling, misleading references, and technical confusion
   - A TG working on this issue must also ensure that technical solutions do not prioritise technical convenience over coherent and unambiguous standard specifications.

While these recommendations and standards are being developed the Task Group recommends that these “dummy” IRIs are kept internal and inline documentation clearly states expectations around their maintenance and resolvability.

### Representing replicates and derived samples/specimens and the relationships between them

As noted in [Issue 24](https://github.com/tdwg/gbwg/issues/24) of this TG’s proceedings, both DwC and MIxS require advancement to reliably and clearly relate replicates (be they technical [i.e. generated to verify stable signals during downstream processing of a sample], or elements of a sampling/experimental design) to one other using metadata fields.

Participants noted that community portals or other users of both standard specifications, rather than the standards bodies themselves, are defining links between replicates and derived samples/specimens. The interoperability of these solutions is questionable. A subsequent TG should investigate how to reconcile and render these interoperable by further coordination between DwC and MIxS, and other specifications in the TDWG and GSC scope.

Further, participants noted that several working groups have explored potential models for this task, and we recommend the subsequent TG should engage them.

Resources:

- <http://www.obofoundry.org/ontology/ro.html>
- <https://www.tdwg.org/community/interaction/>
- <https://www.tdwg.org/community/cd/>
- Droege et al. (2016): The Global Genome Biodiversity Network (GGBN) Data Standard. Database baw125 doi: [10.1093/database/baw125](https://dx.doi.org/10.1093%2Fdatabase%2Fbaw125)
- DwC Term relationshipOfResource: <https://github.com/tdwg/dwc/issues/194>

### Recommendations for richer data exchange formats beyond DwC-A

The widely-used Darwin Core Archive (DwC-A) format arranges data into a simple "star schema" containing core records, such as species occurrences which can be extended in a many-to-one manner, such as multiple images for the occurrence. With no ability to relate records across extensions, the only feasible arrangement is to use an Occurrence core, with an extension that holds the sequence metadata supporting the claim of species occurrence. This is the same conclusion that the GBIF *DNA-related data* task group documented^^[[3]](#footnote-3)^^.

The use of Occurrence core, limits the ability to easily track sampling event data such as arranging a hierarchy of nested samples, and forces unnecessary repetition of data in the DwC-A. In practice, this poses limitations, such as:

1. Metabarcoding of sediment samples, sediment parameter MeasurementOrFact records (porosity, grain size, solutes, organic carbon) will need to be repeated for every occurrence as there are no events to link to
2. Sediment or plankton samples being processed with microscopy, but subsamples are used for metabarcoding. In this case there's no way to indicate parent child relationship between samples and subsamples.

The task group recommends that work is done to explore more expressive data exchange formats ensuring lossless exchange is easily possible.

# Appendix 4

## Relation of interoperable standards to the future of data-driven publishing

Standards alignment can facilitate data and metadata exchange between infrastructures and during the academic publishing process. In fact, the DwC-A format has already been used to exchange biodiversity information across different aggregators using Scratchpads user communities. In addition, DwC Archives have been actively used as supplementary data files associated with research papers in journals such as Zookeys to enrich traditional publications with structured data and for liberating structured content from journals by Plazi. Reuse of standard-compliant biodiversity metadata within the publishing process has also been realised for the Ecological Metadata Language (EML) which can be imported directly into a data paper manuscript from various sources (GBIF, LTER, DataONE). More recently, a workflow for import of genomic metadata from European Nucleotide Archive (ENA), BioSamples and ArrayExpress, part of which is MIxS-compliant, in the narrative of an omics data paper manuscript has also been developed.

All of these advancements in semantic publishing and data exchange signify that the proposed DwC-MIxS mapping would further improve the interoperability between infrastructures and facilitate the reusability of omics and biodiversity metadata. Improved interoperability between standards could also drive scholarly publishers and database managers to implement workflows for standard-compliant data reuse.

1. Within the GOOS EOV framework, methods identified by the GOOS Expert Panel to deliver superior results for any given EOVs, will be endorsed in the UNESCO Ocean Best Practices System (OBPS). To know whether a given measurement value can be used to report on an EOV, information on the method used will be essential, and will thus need to be included as part of the metadata. [↑](#footnote-ref-1)
2. [↑](#footnote-ref-2)
3. <https://doi.org/10.35035/doc-vf1a-nr22> [↑](#footnote-ref-3)
